# AlvaQSAR: A Web-Based Platform for Molecular Prediction of Environmental and Toxicological Endpoints Aligned with OECD Principles

**DOI:** 10.3390/ijms27219787

**Published:** 2026-10-31

**Authors:** Andrea Mauri, Matteo Bertola

**Affiliations:** Alvascience Srl, Via Giuseppe Parini, 35, 23900 Lecco, Italy

**Keywords:** drug discovery, QSAR, cheminformatics, toxicity prediction, OECD regulatory assessment

## Abstract

AlvaQSAR is a web platform for quantitative structure-activity/property relationship (QSAR/QSPR) prediction designed to support rapid molecular screening together with the transparency needed for scientific and regulatory use. The platform combines browser-based molecular input, endpoint selection, prediction, applicability-domain evaluation, and downloadable reporting within a compact workflow, while relying on the Alvascience software ecosystem for molecular standardization, descriptor calculation, and model execution. In version 1.2, the platform provides access to nine models covering environmental fate, bioaccumulation, aquatic toxicity, and human toxicity endpoints, and complements predictions with supporting information such as molecular properties, model documentation, prediction report, and QSAR Model Reporting Format (QMRF) files to address key OECD-oriented expectations on defined endpoints, unambiguous algorithms, validation, and applicability-domain assessment. It aims to facilitate practical use of QSAR models for early screening, communication, and regulatory assessment.

## 1. Introduction

The quantitative structure-activity relationship field (QSAR) has evolved into a mature discipline that is now widely practiced in academia, industry, and for regulatory purposes by government agencies [1,2]. Rapid and reliable QSAR prediction has become particularly important in drug discovery and safety assessment, where substances must be screened early to reduce experimental cost and time while limiting animal testing whenever possible.

In practice, the usefulness of a QSAR model depends not only on predictive accuracy but also on whether it is possible to judge the applicability of the model to the query substance and understand the basis of the prediction. Therefore, the best practice combines predictive performance with applicability-domain analysis [3,4], transparent reporting, and sufficiently rigorous documentation to support scientific and regulatory decision-making.

In regulatory and scientifically rigorous QSAR practice, the Organisation for Economic Co-Operation and Development (OECD) principles [5] provide the core expectations for model validity, including a defined endpoint, an unambiguous algorithm, a defined applicability domain, appropriate measures of goodness-of-fit, robustness and predictivity, and, where possible, a mechanistic interpretation. Their role became especially important in the context of the European REACH regulation, where QSAR models could support chemical assessment in the absence of experimental data only if the validity of their predictions was guaranteed through accurate checks [6]. Similar regulatory frameworks have also been adopted in other jurisdictions, such as Turkey through the KKDIK regulation and the United Kingdom through UK-REACH. Within this perspective, a QSAR Assessment Framework (QAF) has been introduced to help translate those principles into practical evaluation of model predictions by combining model transparency, applicability-domain evidence, and documentation suitable for communication and review. In practice, this means that a prediction should be accompanied by structured supporting information such as the QSAR Model Reporting Format (QMRF), so that the result is not just available but also interpretable and assessable in a regulatory context.

Several software solutions have been developed to facilitate the application of QSAR models for the prediction of physicochemical, environmental, and toxicological endpoints. These include standalone desktop applications, such as VEGA [7] and QSARINS-Chem [8,9], as well as web-based platforms that allow predictions without requiring local software installation. Among the latter, the Danish (Q)SAR Database [10] provides access to a large collection of predictions for existing chemicals, while its associated website allows users to submit new structures and obtain predictions accompanied by applicability domain assessment and supporting documentation. Also, ADMETlab 3.0 [11] offers extensive coverage of absorption, distribution, metabolism, excretion, and toxicity (ADMET) endpoints, together with uncertainty estimation, although model-specific QMRF documentation does not appear to be provided. These platforms demonstrate the availability of established QSAR tools with complementary functionalities and different levels of model documentation, endpoint coverage, and regulatory orientation.

The increasing availability of QSAR platforms reflects the growing importance of computational tools in chemical safety assessment, including their potential use in regulatory processes. In this context, we developed alvaQSAR, a user-friendly web platform accessible at https://www.alvascience.com/alva/qsar/ (accessed on 23 September 2026), designed to unify molecular prediction, applicability-domain evaluation, and documentation-oriented outputs within a single user workflow. The platform internally relies on software components from the Alvascience suite to perform molecular descriptor calculations and generate QSAR/QSPR model predictions [12]. A distinctive value proposition of alvaQSAR is that it aims to couple model output with reportable evidence such as prediction reports and QMRF documentation, which addresses the broader demand for transparent, explainable, and communication-ready QSAR results.

## 2. Results and Discussion

### 2.1. Platform Overview

The alvaQSAR software is implemented as a web platform designed to provide direct access to predictive QSAR models through a guided and compact user workflow. The first version of the software was released in 2025, while version 1.2, described in this paper, expands the coverage of endpoints, adds molecular properties and visualization charts, and improves the supporting documentation, including the QMRF.

The technical architecture combines a browser-based user interface with server-side prediction components (Figure 1). Molecular input is handled in the web interface either by providing a SMILES string or through Ketcher [13], which allows the user to draw a compound directly in the browser. After the user submits the molecule and selects the endpoint of interest, the input data are sent to the Alvascience server, where alvaDesc [14] and alvaRunner are used to process the structure, calculate the molecular properties required by the model, and generate the corresponding prediction. The resulting prediction and associated molecular properties are then returned to the user and displayed in the browser. The platform also allows the user to download a PDF prediction report to support results traceability, communication, and later review.

All the models were prepared by Alvascience using alvaMolecule for data curation, alvaDesc for descriptors and fingerprints (FP) calculation, and alvaModel for model building and validation [12]. Each model is accompanied by a lightweight HTML documentation accessible through the alvaQSAR platform, while a more complete model description was also prepared in QMRF format.

The platform is designed to support the main OECD principles as follows: a defined endpoint is reflected in the endpoint-based organization of the user interface; an unambiguous algorithm is supported through the associated model documentation and QMRF; the applicability domain is explicitly reported together with the prediction; the goodness-of-fit, robustness, and predictivity of the models are documented in the QMRF.

### 2.2. Workflow

The user workflow in alvaQSAR can be described in four main steps (Figure 2). First, the user defines the molecular structure of interest. This can be done by drawing the compound directly in the browser-based molecular editor or by entering a SMILES string. Both input modes are supported to give users flexibility.

If the submitted structure is invalid or falls outside the scope of the implemented models, such as disconnected molecules (e.g., salts or containing individual ionized atoms), inorganic or organometallic compounds, the platform notifies the user, and no prediction is performed.

Second, the user selects the predictive model associated with the endpoint of interest. The platform presents the available endpoints as a set of cards, each with a name, description, and icon corresponding to environmental or toxicological tasks. This layout allows users to move quickly from molecule definition to model prediction.

Third, the user can analyze the generated output. The prediction itself is the core result, but it is accompanied by information intended to support interpretation. One of the most important elements is the applicability domain: the prediction can be classified as inside the applicability domain (IN) or outside it (OUT), helping the user understand whether the query compound falls within the model space and whether the result can be considered reliable. Additional information related to the input molecule, such as its properties, is displayed in both tabular and graphical formats.

Fourth, the user can download a prediction report in PDF format. This report is intended to provide a persistent summary of the query, the selected endpoint and model, the produced result, and the related applicability domain information. In practice, this export capability is important for internal documentation or in contexts where the prediction must be communicated as part of a broader assessment record.

Overall, the workflow is intentionally compact and user-friendly. By reducing the operational distance between molecular input and documented result, alvaQSAR enables a rapid screening experience.

### 2.3. Use Case

As a use case, alvaQSAR has been used to predict the ready biodegradability and eye irritation for 3-chlorobenzoic acid (CAS RN 535-80-8), which is an organic compound. It was selected as a representative external substance because it is absent from both the ready biodegradability and eye irritation datasets.

For ready biodegradability, the compound was correctly predicted as ready biodegradable [15] within the applicability domain but with low reliability, reflecting the presence of structural features associated with lower biodegradability, such as an aromatic ring and halogen substitution, together with a carboxylic acid group, which is commonly associated with enhanced biodegradability [16].

The eye irritation model properly predicted the compound as an irritant [17] with high reliability and within the applicability domain. The alvaQSAR report includes the prediction result together with reliability and applicability domain information, helping users assess the quality of the prediction (Figure 3).

## 3. Methods and Materials

### 3.1. Data Curation

Nine endpoints related to environmental fate, bioaccumulation, aquatic toxicity, and human toxicity can be predicted using alvaQSAR (Table 1).

The datasets used to develop the models included in alvaQSAR have been taken from different sources [18,19,20,21,22,23,24]. Even if all data taken from the literature were already curated by the authors of the papers, prior to model development, additional data curation and structure standardization were performed to ensure endpoint consistency and structural reliability [25,26]. Data curation has been performed using alvaMolecule.

In the dataset for 48-h LC50 values for Daphnia magna [21,23] and 96-h LC50 values for fathead minnow, no inconsistencies have been found. For all other datasets, some molecules have been removed.

In most datasets, some organometallic compounds have been removed; e.g., 13 organometallic molecules including cobalt, tin, and zinc have been identified and removed from the bioconcentration factor dataset [19].

Additionally, molecule removal has been performed if no correspondence was found between the CAS RN value, provided by the original authors, and those obtained from PubChem starting from the original SMILES.

Eye irritation dataset [24] consistency was further cross-checked against an external dataset [17]; a total of 158 molecules having different eye irritation values between the two sources have been removed.

### 3.2. Model Preparation

Among the nine available models, four classification models have been developed from scratch using data taken from the STopTox datasets [24] (acute oral toxicity, skin irritation, eye irritation and skin sensitization), while five of them are based on models presented in the literature. Among these, the ready biodegradability model was modified by removing the F01[N–N] descriptor from the pool of 12 descriptors proposed by the authors [18] due to its quasi-constant behavior in the curated dataset.

The same workflow for model development has been applied to all four newly developed classification models. As a first step, an initial pool of 2-dimensional molecular descriptors and fingerprints has been calculated using alvaDesc [14].

Subsequently, model development was performed within alvaModel. The curated datasets were split into training (60%) and test (40%) sets before modeling using a Venetian Blind splitting procedure. The test sets preserve the class proportion observed in the full datasets.

The training set was used to choose the best modeling approach, select the relevant molecular descriptors, and obtain the final models. The test set served only to validate the final pools of models.

Prior to feature selection, the number of molecular descriptors was subjected to preprocessing to remove:

1. Constant and near-constant descriptors
2. Highly correlated descriptors [27]
3. Descriptors with missing values

Feature selection was carried out using a Genetic Algorithm (GA) optimization approach [28]. The GA was applied to identify minimal subsets of descriptors that maximize predictive performance while minimizing redundancy and overfitting.

The final descriptor subset was selected based on internal cross-validation performance, model stability, and classification accuracy criteria.

Additionally, a Y-randomization procedure involving 500 permutations of the response variable was performed during model development to assess the risk of chance correlations and evaluate whether the observed predictive performance could be attributed to random associations between molecular descriptors and the response variable.

All models implemented in alvaQSAR are provided with a properly defined applicability domain, indicating whether a predicted substance falls within the chemical space of the model [3,4].

In addition to the applicability domain information, classification models’ predictions are coupled by a reliability level. Reliability is derived from the class probability estimated by the considered model. Predictions with probabilities far from the decision threshold are assigned higher reliability, while probabilities close to the threshold are assigned lower reliability. Distribution of reliability levels in the training set has been used as a further rule for model selection.

Reliability classification is independent of the applicability domain assessment and can be used as additional information in combination with the AD results.

After completion of external validation, the final models were retrained on the full datasets prior to deployment in alvaQSAR. The recalibration, using the entire set of experimental data, allows full exploitation of the experimental information.

### 3.3. Available Models

The alvaQSAR platform currently includes nine different models (Table 2 and Table 3).

The available models can be used for regulatory screening, environmental risk, and PBT assessment by analyzing chemical structures to estimate safety without animal testing.

The environmental fate endpoint included in alvaQSAR is estimated by the ready biodegradability model, which is a classification model that predicts whether a chemical degrades rapidly under aerobic conditions or not. It can help identify environmentally persistent substances. Experimental values have been collected following the OECD Test Guideline 301C and measure biochemical oxygen demand (BOD) in an aerobic aqueous medium over 28 days. The model is derived from the k-nearest neighbors (kNN) model [29,30] described by Mansouri et al. and is based on 11 molecular descriptors calculated from standardized 2D molecular representations.

Bioaccumulation can be explored using the bioconcentration factor (BCF) and biomagnification factor (BMF) models. Both are regression models that can be used for the evaluation of persistence, bioaccumulation, and toxicity assessment. The bioconcentration factor in aquatic organisms is expressed as the ratio between the concentration of a chemical in aquatic organisms and its concentration in the surrounding water at a steady state. The model is based on the PLS model described by Bhattacharyya et al. and uses 2D molecular descriptors. To improve interpretability, alvaQSAR provides the predicted BCF value and a label for bioaccumulative and non-bioaccumulative compounds, considering that a substance fulfils the bioaccumulation criterion when the bioconcentration factor in aquatic species is higher than 2000 L/kg.

The BMF is defined as the ratio between the concentration of a chemical in fish and its concentration in the organism’s diet. The BMF model implements a published QSAR model based on a consensus prediction approach that combines outputs from a kNN model and an ordinary least squares (OLS) model [20]. Both models rely on numerical molecular descriptors calculated from standardized 2D chemical structures.

The aquatic toxicity endpoints can be estimated using two regression models developed to predict Daphnia magna and fathead minnow acute toxicity. These are two standard organisms used in ecotoxicological assessment of chemicals.

Daphnia magna acute toxicity is expressed as 48-h LC50, i.e., the concentration causing 50% lethality after 48 h of exposure, and is modeled by a consensus model including two kNN models, the first one using 2D descriptors and the second one implementing similarity through extended connectivity fingerprints (ECFP) [21,22].

Acute toxicity toward fathead minnow (Pimephales promelas) is expressed as 96-h LC50 and modelled as −log(mol/L). It is implemented as a kNN model based on 2D molecular descriptors [23].

The ready biodegradability model and the four regression models implemented in alvaQSAR were reproduced from previously published models, and their predictive performances were generally consistent with those reported in the original studies. Specifically, the ready biodegradability model achieved a cross-validated Sensitivity and Specificity of 0.82 and 0.87, respectively, compared with values of 0.84 and 0.88 reported for the kNN model by Mansouri et al. [18]. Among the regression models, the BCF model achieved an R^2^ and Q^2^ of 0.68 and 0.67, respectively, compared with values of 0.65 for both R^2^ and Q^2^ (LOO) for the PLS-QSPR model reported by Bhattacharyya et al. [19]. The BMF model achieved an R^2^ and Q^2^ of 0.79 and 0.77, respectively, compared with 0.81 and 0.79 reported for the original consensus model [20]. The Daphnia magna consensus model achieved an R^2^ and Q^2^ of 0.64 and 0.65, compared with 0.60 and 0.61 for the first kNN model, and 0.78 for both metrics of the second kNN model reported by Cassotti et al. [21]. Finally, the acute toxicity toward the fathead minnow model achieved a value of 0.62 for both R^2^ and Q^2^, compared with 0.62 and 0.61 reported for the original model with no applicability-domain threshold [23].

A set of four new classification models covering human toxicity endpoints have been developed. Skin irritation potential is classified as irritant/non-irritant following the Global Harmonization System (GHS); the prediction is performed using a Quadratic Discriminant Analysis (QDA) model based on 2D molecular descriptors.

Eye irritation is a local toxic effect characterized by reversible or irreversible damage to ocular tissues, including the cornea, iris, and conjunctiva, following exposure to a chemical substance. Eye irritation is predicted with a QDA model, including 2D molecular descriptors.

The skin sensitization model predicts sensitization potential from the Local Lymph Node Assay (LLNA). The model provides binary classification consistent with GHS criteria: sensitizer/non-sensitizer. The implemented model is a Support Vector Machine (SVM) classifier [31] that uses molecular descriptors.

The acute oral toxicity model provides binary classification consistent with GHS criteria. A substance is considered toxic if LD50 is less than 2000 mg/kg body weight and not classified otherwise. The developed model is a SVM classifier using 2D molecular descriptors.

Compared with the original STopTox models, the four newly developed classification models showed generally comparable or improved performance in terms of Accuracy, Sensitivity, and Specificity. Specifically, the skin irritation model achieved 0.73, 0.90, and 0.65, respectively, compared with 0.72, 0.77, and 0.66 for STopTox; the eye irritation model achieved 0.78, 0.79, and 0.78 compared with 0.72, 0.72, and 0.71; the skin sensitization model achieved 0.76, 0.81, and 0.71 compared with 0.70, 0.66, and 0.75; and the acute oral toxicity model achieved 0.75, 0.84, and 0.63 compared with 0.77, 0.85, and 0.70.

The classification models were developed using datasets with different degrees of class balance. The skin sensitization dataset is nearly perfectly balanced, with 49.7% sensitizers and 50.3% non-sensitizers, while the acute oral toxicity dataset shows a relatively balanced distribution, with 57% toxic and 43% non-classified compounds. Conversely, the skin irritation and eye irritation datasets exhibit moderate class imbalance, with 32.1% and 34.2% irritant compounds, respectively. To complement the performance metrics reported in Table 2 and provide a more informative evaluation in the presence of class imbalance, the F1 score was also considered. The resulting F1 scores were 0.81 for ready biodegradability, 0.67 for skin irritation, 0.72 for eye irritation, 0.82 for skin sensitization and 0.83 for acute oral toxicity.

All models implemented in alvaQSAR are applicable to organic chemicals for which the model descriptors can be calculated. They are not applicable by design to mixtures, inorganic and organometallic compounds, and those compounds falling outside the defined multivariate descriptor space.

All models are provided with an applicability domain (AD), which defines the region of chemical space represented by the training compounds.

For the BCF model, the AD is defined using a leverage-based approach, which measures the position of a query compound relative to the centroid of the training set in the multivariate descriptor space. The calculated leverage is compared with a threshold determined by the number of training compounds, the number of descriptors, and a predefined threshold factor of 3. Compounds with leverage values below this threshold are considered within the AD, whereas those exceeding it are classified as outside the AD.

For the skin irritation, skin sensitization, eye irritation, ready biodegradability, fathead minnow acute toxicity, and acute oral toxicity models, the AD is assessed using distance-based methods. The distance of each query compound from the centroid of the training set is calculated using the molecular descriptors and distance metric associated with the model. This distance is compared with a model-specific threshold, which is defined using a multiple of 2 of the average distance for the fathead minnow model, and a 95th percentile of the distance distribution of the training compounds for skin irritation, skin sensitization, eye irritation, ready biodegradability, and acute oral toxicity models. Compounds with distances below the threshold are considered within the AD, whereas those exceeding it are classified as outside.

For the BMF and Daphnia magna acute toxicity models, the AD is evaluated as the conjunction (Boolean AND) of the ADs associated with their underlying methods of the consensus models. For models combining multiple predictive components, the overall AD is defined as the intersection of the individual applicability domains, meaning that a compound is considered within the AD only when it satisfies the criteria of all the component models.

## 4. Conclusions

AlvaQSAR provides a practical way to access QSAR/QSPR models through a web interface that reduces the distance between molecular input, prediction, applicability-domain assessment, and report generation. The platform is intended not only to deliver rapid estimates for relevant environmental and toxicological endpoints, but also to make those predictions easier to interpret and communicate by pairing them with molecular properties, model documentation, a prediction report, and a QMRF file. This combination is particularly relevant for screening activities carried out in scientific, industrial, and regulatory settings, where the usefulness of a prediction depends on both performance and supporting evidence. As presented in this manuscript, alvaQSAR extends the Alvascience software suite into an online service and offers an accessible framework for transparent QSAR-based assessment of organic chemicals. The case study included in this manuscript is intended to illustrate how these elements come together in a realistic prediction workflow, from structure submission to interpretation of the reported result.

## Figures and Tables

**Figure 1:**
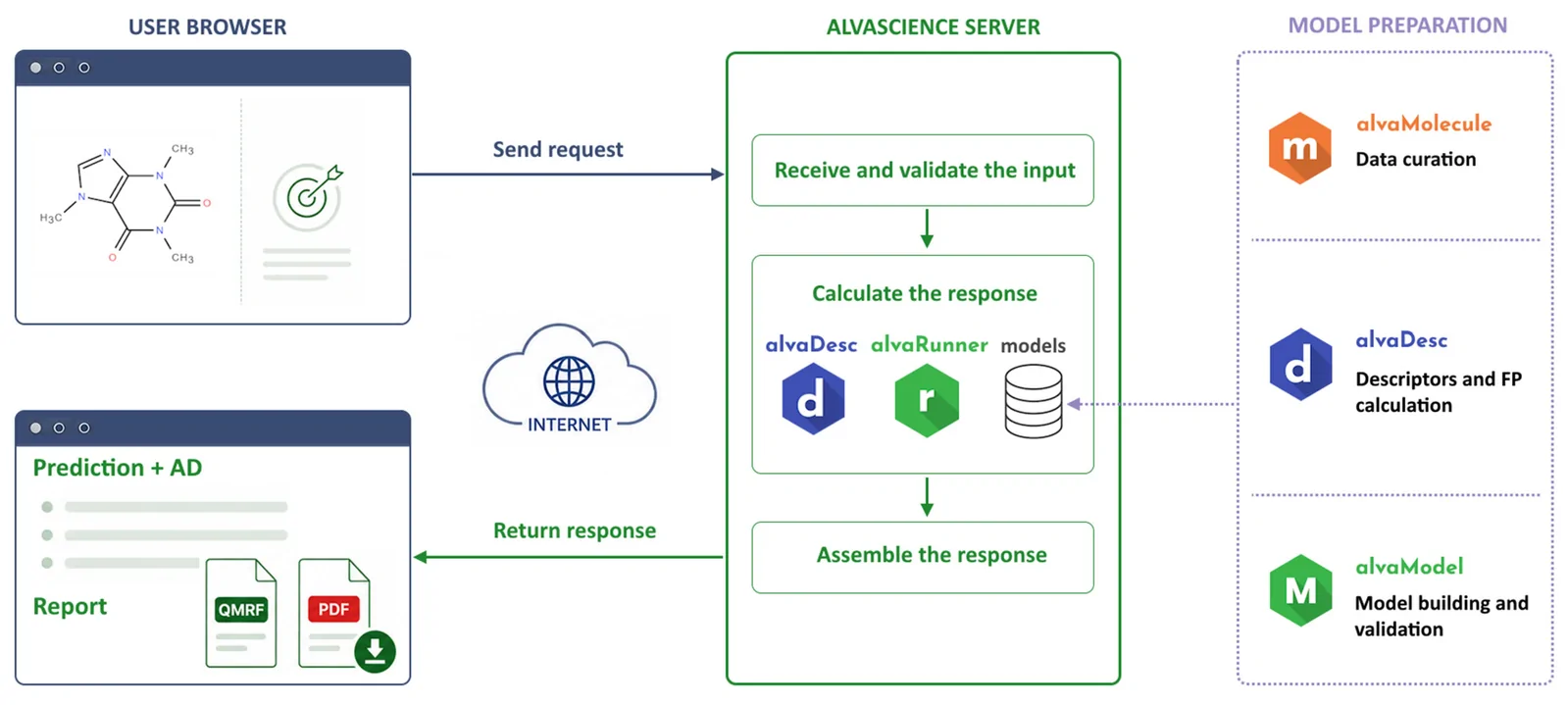
Architecture and data flow of the alvaQSAR platform, including the software tools used for model preparation.

**Figure 2:**
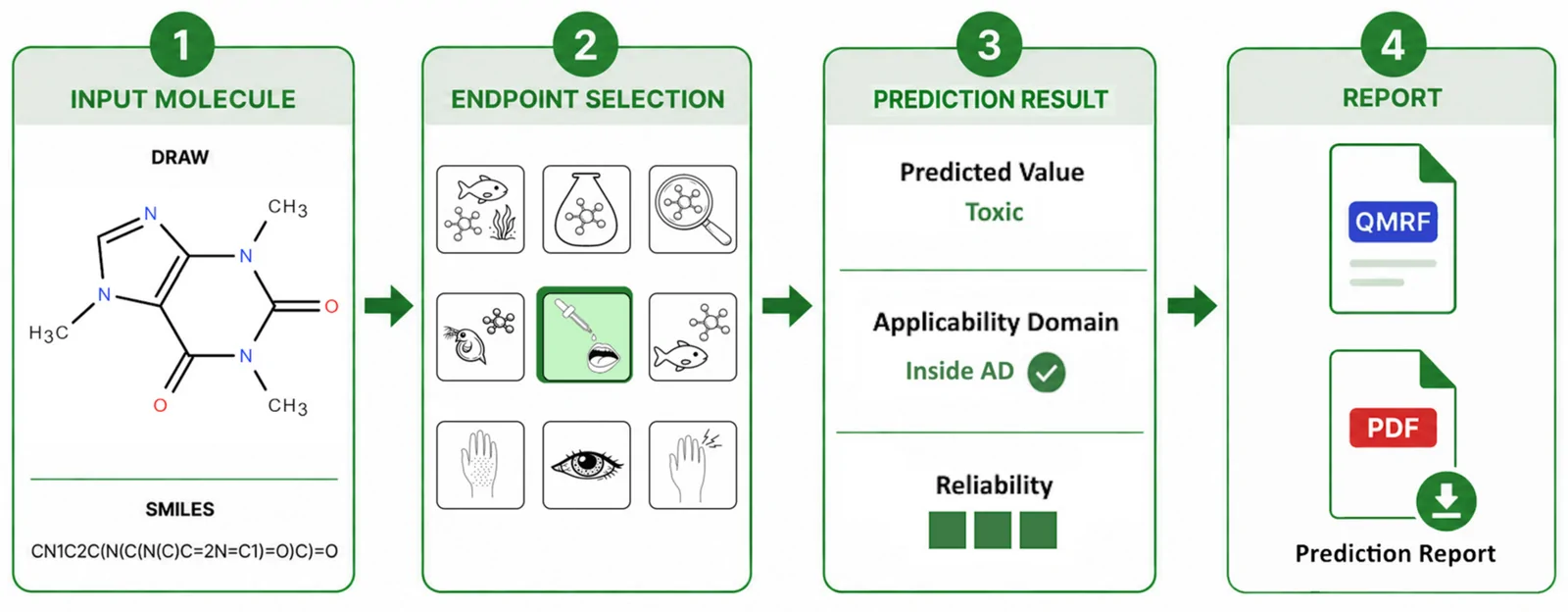
Key steps of the alvaQSAR workflow. The user can input the molecule (Step 1), choose the endpoint of interest (Step 2), check the prediction result (Step 3), and download the prediction report (Step 4).

**Figure 3:**
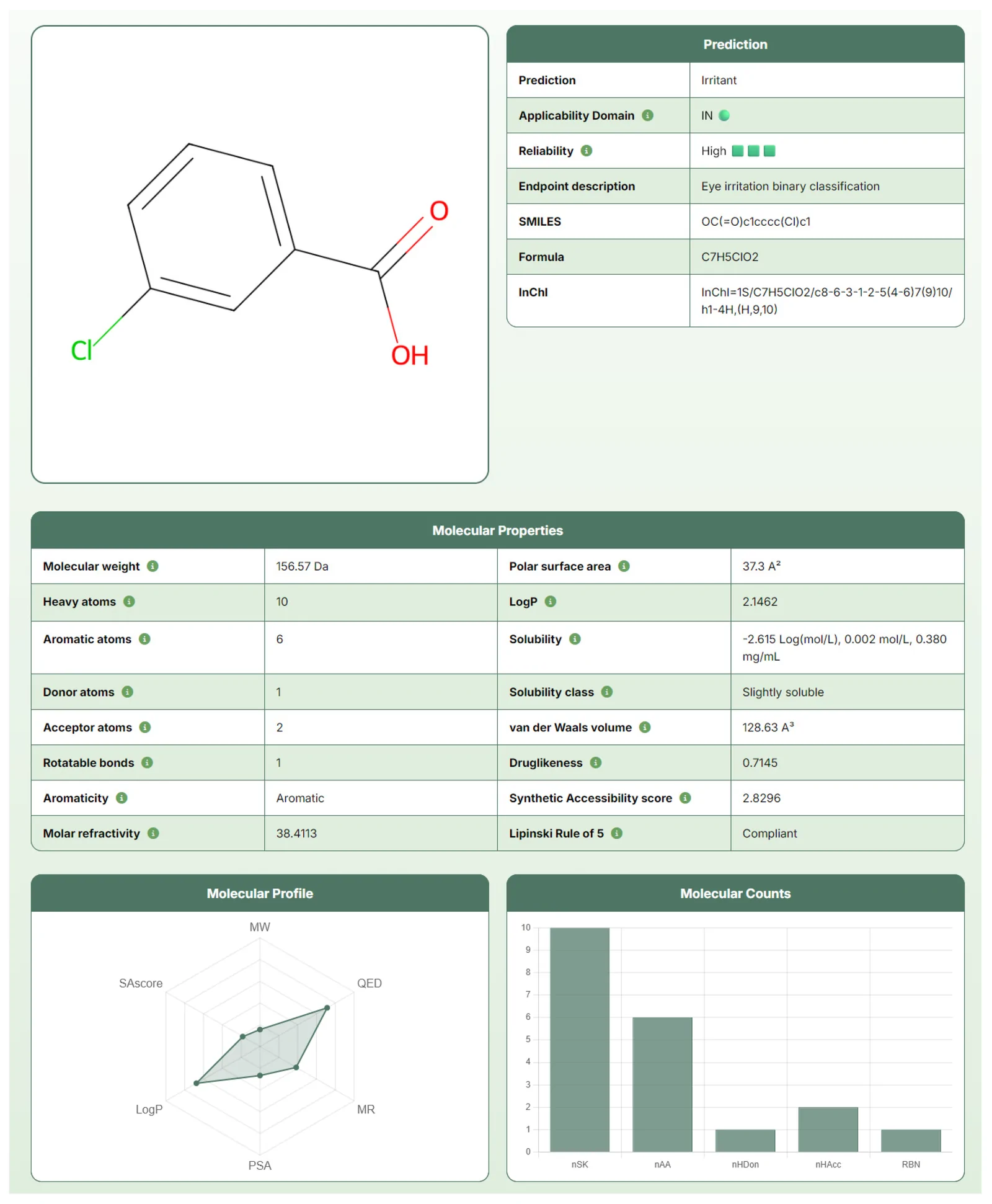
Screenshot of alvaQSAR result page showing the eye irritation model prediction for 3-chlorobenzoic acid.

**Table 1:** List of available endpoints. The column “Compounds” includes the number of molecules in the curated and original dataset. The column “OECD TG” refers to the Organisation for Economic Co-operation and Development Test Guideline.

Endpoint	Category	Model Type	Compounds	Regulation	OECD TG
Ready Biodegradability	Environmental fate	Classification	1003/1056	REACH 9.2.1.1	301C
Bioconcentration Factor (BCF)	Bioaccumulation	Regression	1290/1303	REACH 9.3.2	305
Biomagnification Factor (BMF)	Bioaccumulation	Regression	211/214	PBT/vPvB assessment	305
Daphnia magna Acute Toxicity (48 h LC50)	Aquatic toxicity	Regression	546/546	REACH 9.1.1	202
Fathead Minnow Acute Toxicity (96 h LC50)	Aquatic toxicity	Regression	908/908	REACH 9.1.3	203
Skin Irritation	Human toxicity	Classification	928/1012	REACH 8.1	404
Eye Irritation	Human toxicity	Classification	3029/3545	REACH 8.2	405
Skin Sensitization	Human toxicity	Classification	902/1000	REACH 8.3.2	429/442B
Acute Oral Toxicity	Human toxicity	Classification	8018/8442	REACH 8.5.1	420/423/425

During data curation, the following steps were applied: Removal of duplicated structures; Removal of inorganic substances and organometallic compounds; Removal of substances for which SMILES and CAS RN were not concordant.

**Table 2:** List of endpoints modeled using classification models, including performance metrics (CV: 5-fold cross-validation).

Endpoint	Training			Test		
	Accuracy CV	Sensitivity CV	Specificity CV	Accuracy	Sensitivity	Specificity
Ready biodegradability	0.85	0.82	0.87	0.85	0.81	0.87
Skin irritation	0.73	0.90	0.65	0.70	0.81	0.65
Eye irritation	0.78	0.79	0.78	0.79	0.76	0.81
Skin sensitization	0.76	0.81	0.71	0.73	0.82	0.65
Acute Oral Toxicity	0.75	0.84	0.63	0.75	0.85	0.61

**Table 3:** List of endpoints modeled using regression models.

Endpoint	Training				Test	
	R^2^	RMSE	Q^2^ CV	RMSE CV	R^2^	RMSE
Bioconcentration Factor (BCF)	0.68	0.79	0.67	0.80	0.66	0.83
Biomagnification Factor (BMF)	0.79	0.50	0.77	0.53	0.83	0.45
Daphnia magna Acute Toxicity (48 h LC50)	0.64	0.96	0.65	0.98	0.55	1.11
Fathead Minnow Acute Toxicity (96 h LC50)	0.62	0.88	0.62	0.88	0.65	0.92

## Data Availability

AlvaQSAR is available at https://www.alvascience.com/alva/qsar/ (accessed on 23 September 2026). AlvaQSAR is a proprietary web-based platform developed by Alvascience. Access to the platform is free and requires user registration through the Alvascience website.
